# Sneeze and pop: a ruptured varicocele; analysis of literature, guided by a well-documented case-report

**DOI:** 10.1186/s12894-019-0442-z

**Published:** 2019-01-31

**Authors:** Daan J. Reesink, Peter M. Huisman, Judith Wiltink, Arto E. Boeken Kruger, Tycho M. T. W. Lock

**Affiliations:** 1 Department of Urology, Tergooi Hospital Hilversum, Rijksstraatweg 1, 1261 AN Blaricum, The Netherlands; 2 Department of Radiology, Tergooi Hospital, Rijksstraatweg 1, 1261 AN Blaricum, The Netherlands; 3 Department of Urology, University Medical Center Utrecht, Heidelberglaan 100, 3584 CX Utrecht, The Netherlands

**Keywords:** Idiopathic spontaneous varicocele rupture, Spermatic cord hematoma, Hematocele

## Abstract

**Background:**

An acute scrotal hematoma, secondary to a spontaneous rupture of a varicocele is still a rare presentation in daily practice. However, multiple case reports have been reported. Sudden increase in abdominal pressure, resulting to an increased venous pressure can lead to a rupture of the varicocele. Literature search shows that due to uncertainty of the diagnosis, explorative surgery is often performed, sometimes resulting in unnecessary orchiectomies. The objective of this study was to determine classical clinical presentation of patients with a spontaneous rupture of a varicocele, determine the diagnostic procedure, and give an insight in the follow-up.

**Case presentation:**

We present a case of a 24-year old male with acute scrotal swelling after sneezing. Subsequently, we carried out a systematic literature search to identify all eligible studies to determine classic clinical presentation of spontaneous ruptures of a varicocele.

**Conclusion:**

The literature search shows that clinical presentation of idiopathic spontaneous scrotal hematomas is similar to testicular torsion, epididymo-orchitis, malignancy, or (incarcerated) inguinal hernia making differential diagnosis difficult. Especially when there has been increased abdominal pressure or strenuous activity preceding the symptoms, and the swelling is left sided, it should be included in the differential diagnosis for patient with acute inguinoscrotal swelling. Colour Doppler-Ultrasonography is recommended to distinguish between other causes of acute scrotum. The hematoma is usual self-limiting, justifying conservative treatment. Early surgical intervention is indicated with signs of ischaemia due to obstruction, infection of the hematoma, or uncertain diagnosis (i.e. malignancy). However, physicians should be cautious with direct exploration, as it led to unnecessary orchiectomy in 25% of patients. The hematoma can increase in size up to 3 months post-event, and it can take up to 15 months to completely resolve.

## Background

A varicocele is present in about 15% of healthy males [1, 2]. A spontaneous rupture of a varicocele, resulting in an acute scrotal hematoma however, is a rare phenomenon. Sudden increase in abdominal pressure, resulting to an increased venous pressure can lead to a rupture of the varicocele. Symptoms can be similar to a torsion of the testis, torsion of appendix testis, epididymo-orchitis or malignancy. Imaging has shown to be challenging. We present a case of a 24-year old male with acute scrotal swelling.

A literature search on this topic during treatment of this patients resulted in the finding that due to uncertainty, explorative surgery is often performed, sometimes resulting in unnecessary orchiectomies. The aim of this study was to determine classical clinical presentation of patients with a spontaneous rupture of a varicocele, determine the diagnostic procedure and give an insight in the follow-up.

## Case presentation

**Fig. 1:**
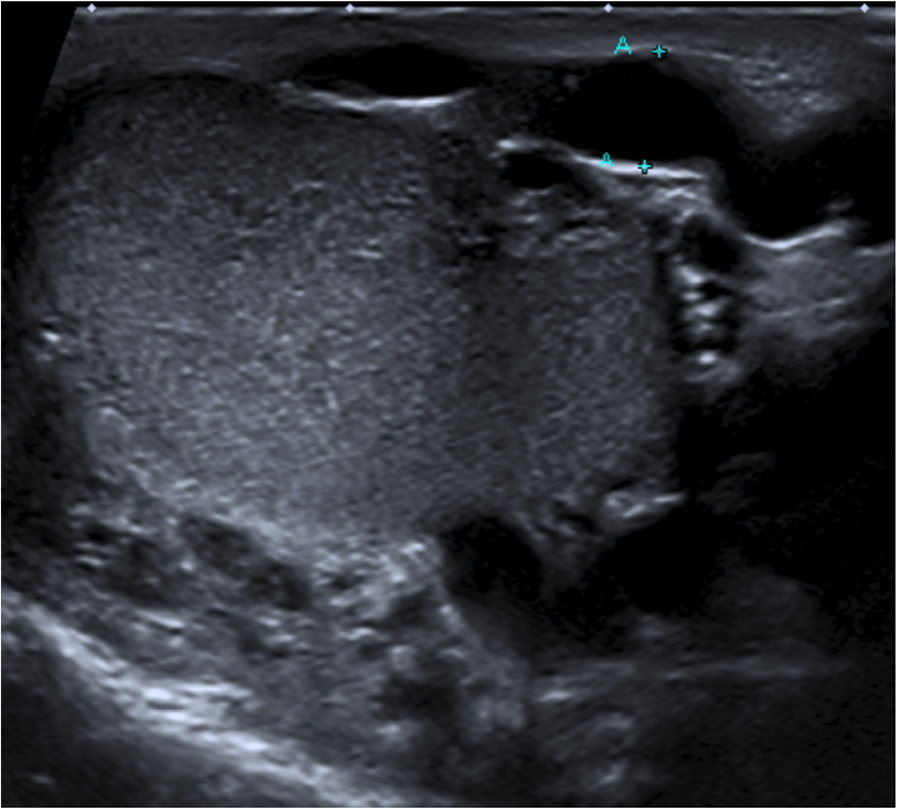
Ultrasound of the left testis, 5 months prior to the ER visit, during a Valsalva Manoeuvre. A clear varicocele of 4.3 mm is visible (A-A)

**Fig. 2:**
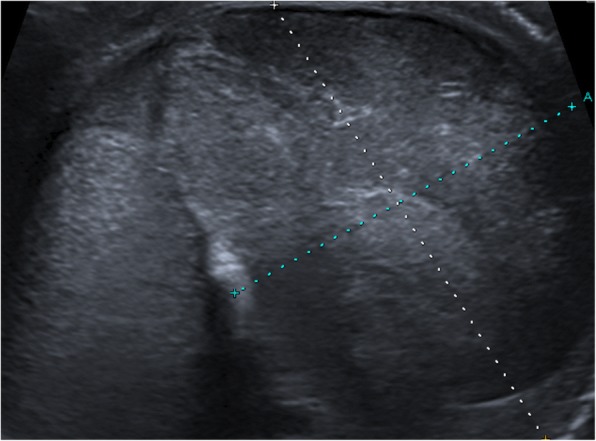
Ultrasound of the same testis left, after presentation at the ER. Lateral of the testis, a large hypo echogenic, non-vascular mass of 39×29mm is visible

**Fig. 3:**
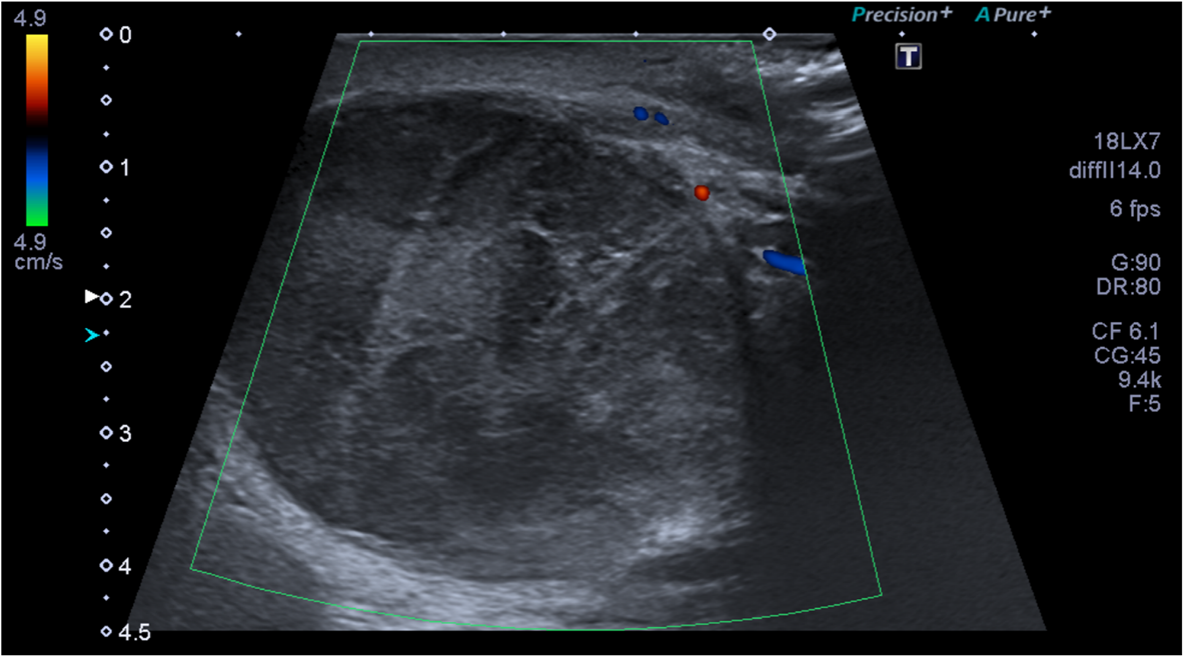
Colour Doppler Ultrasound of the same testis left, 2 months after presentation. There is progression of the size of the mass to 40×40mm. The swelling is not demarcatable from the left testis. Although there is no vascular flow visible, due to the progression in size and the aspect of the swelling, a malignancy cannot be ruled out

**Fig. 4:**
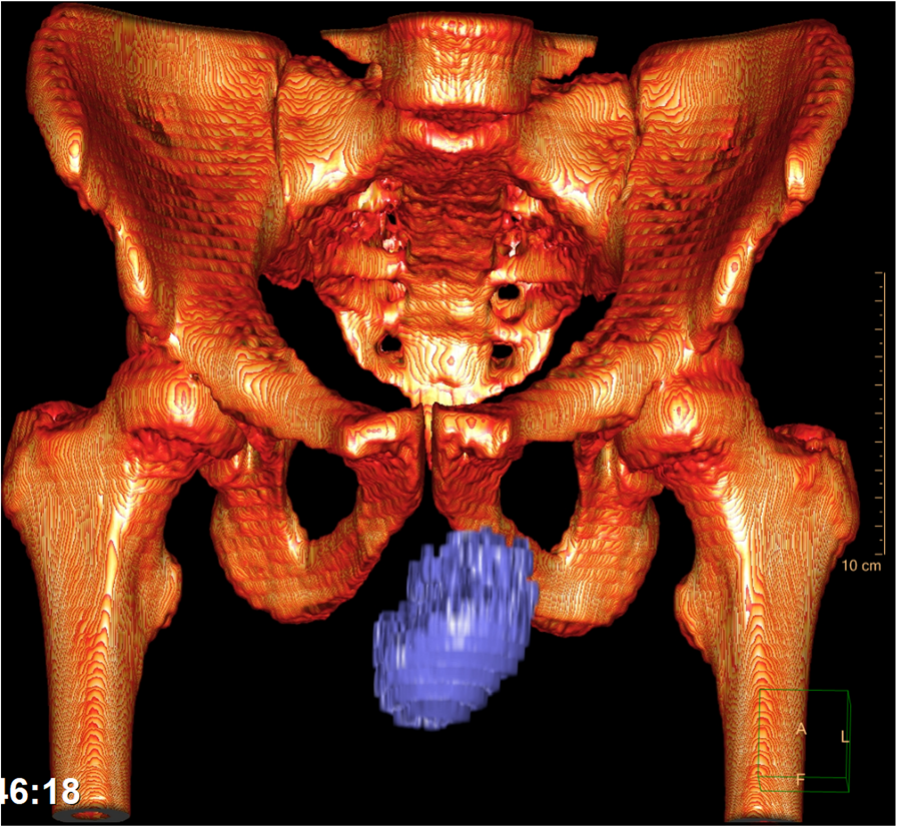
Coronal 3D-replication of the CT images, illustrating the size of the hematoma

A 24-year old male was seen at the Emergency Department of our hospital with acute scrotal swelling on the left side, which started 5 days earlier. The symptoms started during a trip to Japan, where the patient had multiple severe sneezes while walking outside. On examination, he had a large swelling of the left hemiscrotum. Except for a left sided varicocele (Fig. 1), which was diagnosed 6 months earlier in our hospital, the patient had no medical history. Blood-results were negative. Colour Doppler-Ultrasonography (CDU) showed the known varicocele, a normal vascularized left testis, and a swelling of low echogenicity of 39x29mm without blood flow, suiting a scrotal bleeding (Fig. 2). The hematoma was considered self-limiting, and spontaneous resorption was expected. However, after follow-up ultrasonography 2 months later, the swelling had increased in size (40x40mm) (Fig.3). The patient was referred to an academic hospital. A CT-scan of the abdomen showed a prominent vena spermatica on the left, without suspicion of an arteriovenous malformation. A 3D replica of the CT-scan, illustrating the size of the hematoma (Fig. 4).

Three months post-event, the hematoma even further increased in size to 50x37x30mm. Eventually, the patient underwent a microscopic inguinal varicocelectomy. After, the hematoma showed signs of reabsorption, decreasing in size to 38x24x21mm 4 months; 20x16x11mm 6 months; and to no residual hematoma eventually, 15 months post-event. The left testis itself did not differ in size at all follow-up points.

## Discussion

An acute scrotal hematoma, secondary to a spontaneous rupture of a varicocele is still a very rare presentation in daily practice. However, multiple case reports have been reported.

### Literature search

We carried out a systematic literature search for PubMed, EMBASE, and Cochrane Library, using Medical Subject Headings indexes, keyword searchers and publication types until May 2017. Additionally, the reference lists of included articles/case reports were also examined for potential studies. There was no language restriction, as long as all relevant info was present (in the abstract), and the abstract was in English. The following keywords were used in the search: varicocele rupture, or spermatic cord hematoma, or hematocele spermatic cord. The terms were separately searched or connected by the Boolean operators “AND” and “OR”. We identified in our search 31 case reports. Differentiation could be made based on etiology into three major causes, i.e. idiopathic, spontaneous hematomas (18×) [3–20], (direct) traumatic (6×) [21–26], as a result of a coagulation disorder (4×) [27–30]. No abstract or not all necessary information was available in seven case reports. Since this article is on idiopathic, spontaneous hematoma’s, only these case reports will be discussed. Results of the literature search are shown in Table 1.

**Table 1:** Overview of case reports on idiopathic, spontaneous scrotal hematomas

Article/Case Report (year)	Age (years)	Preceding cause	Side (L/R)	Symptoms	Conservative or Treatment	Varicocele present?
Akay et al. (2015)	21	After marching	R	Pain, swelling, ecchymosis	Delayed exploration & orchiectomy due to ischaemia	
Aliabadi et al. (1987)	27	After defecation	L	Pain, swelling, ecchymosis	Exploration due to uncertain diagnosis	Yes
Bowman et al. (1998)	23	After football tackle	R	Pain, swelling, ecchymosis	Conservative.	
Chin et al. (2009)	33	After heavy lifting	L	Pain, swelling	Delayed exploration due to no improvement	Yes
Demir et al. (2010)	21	After defecation	L	Pain, swelling, ecchymosis	Conservative. Ligation after 3 weeks	Yes
Gordon et al. (1993)	22	After blunt abdominal trauma	L	Pain, swelling	Exploration due to uncertain diagnosis	Yes (History)
Kampel et al. (2015)	?	After centrifuge training	L	Pain, swelling	Conservative.	Yes
Kobayashi et al. (2006)	28	After defecation	L	Pain, swelling	Conservative. Ligation after 4 months	
Lindhorst et al. (2000)	53	After playing saxophone	L	Pain, swelling	Exploration due to suspected incarcerated inguinal hernia	Yes
Matsui et al. (2004)	69	After defecation	L	Pain, swelling	Conservative.	
Mirilas et al. (2010)	10	After skiing	L	Pain, swelling,	Exploration due to persisting pain	
Miyoshi et al. (1980)	22	?	L	Pain, swelling, ecchymosis	Exploration due to uncertain diagnosis.	
Nishiyama et al. (2005)	23	After sexual intercourse	L	Pain, swelling, ecchymosis	Conservative	Yes
Pepe et al. (2015)	16	After blunt abdominal trauma	L	Pain, swelling	Exploration due to persisting pain	
Ragozzino et al. (1993)	40	After stretching		Pain, swelling	Conservative	
Rolnick et al. (1965)	16	?	R	Pain, swelling, ecchymosis	Exploration & orchiectomy due to uncertain diagnosis	
Takezawa et al. (2011)	31	Secondary to nutcracker phenomena	L	Pain, swelling	Conservative	Yes
Vandana et al. (2015)	71	Unknown	L	Pain, swelling	Exploration & orchiectomy due to uncertain diagnosis	

L = Left, R = Right.? = Unknown. Symptoms with strikethrough indicates symptom not present

### Etiology

Idiopathic, spontaneous hematomas are thought to be the result of sudden increase of abdominal pressure transmitting to a varicocele. It’s arbitrary whether abdominal trauma can lead to increased abdominal pressure, resulting in a ruptured varicocele [8]. A study of Shafik & Bedeir (1980)*.* showed that patients with a left-sided varicocele develop an increased venous pressure during a Valsalva manoeuvre [31]. Various structures, (i.e. vein of the normal pampiniform plexus, a single varix or false aneurysm of the spermatic artery), have been identified as bleeding source. However, in the majority of cases the bleeding source at surgery cannot be identified [10].

### Clinical presentation

Average age of presentation was 31 (±18) years, with the overall majority of males in their 20s at presentation. This makes distinction between possible testicular torsion, or malignancy difficult, since both affect mostly males between 15 and 35 [32]. Accurate differential diagnosis can possibly be facilitated by taking the patients history with emphasis on possible triggering events. Preceding events to the spontaneous scrotal hematoma in literature could all be narrowed down to increased abdominal pressure. Existing literature describes various activities preceding a spontaneous scrotal hematoma, reviewed in Table 1. Examples are pressure during defecation [4, 7, 9, 11], sexual intercourse [13], blunt abdominal trauma [5, 8, 14], heavy lifting [6], stretching in tight pants [15] or after fighter-pilot centrifuge training [19]. Another patient had a spontaneous hematoma after playing the saxophone [10]. After an inguinal hernia correction at 18 years, doctors had discouraged him from playing ever again. Although strict compliance of this advice, 35 years later he could not resist the urge to play. He presented himself at the ER an hour later. To our knowledge, a scrotal hematoma resulting from sneezing has never been described. When an unknown swelling is present in the scrotum, physicians should include the question whether any activities increasing abdominal pressure, or any strenuous activities, had preceded the swelling, when taking patients history.

Reported clinical presentation of patients with a spontaneous ruptured varicocele is acute pain (83%), and acute (inguinal) scrotal swelling (100%), which could also be difficult to distinguish from testicular torsion, appendix testis torsion, or malignancy. Important to note is that patients with testicular cancer commonly present with a painless mass. Just 10 % of patients present with acute symptoms such as pain [33], so when pain is present malignancy becomes more unlikely. Ecchymosis can sometimes be seen after a few days (33%), but is rarely reported on. Unfortunately, none of the case reports reported on the cremaster reflex. With the exception of three cases, all spontaneous hematoma’s (83%) were found on the left side, conform the higher incidence of left sided varicocele [2]. Varicoceles are more common on the left side due to a longer left testicular vein and congenital incompetence of the valves of the left testicular veins [34]. Other causes are entering of the left testicular vein on the left renal vein at a right angle, arching of the left testicular vein over the left testicular artery thereby compressing it (in some men), compression of the left testicular vein by the descending colon, or compression of the left renal vein between the superior mesenteric artery and the abdominal aorta (nutcracker effect) [35]. The question arises whether the right-sided cases therefore should be categorized as traumatic, even though their described etiology was different.

### Diagnostics

Diagnosing a cause of acute scrotal pain and swelling can be difficult, and as stated the differential diagnosis should include conditions such as testicular torsion, malignancy, epididymo-orchitis, or (incarcerated) inguinal hernia [6, 14].

Blood analyses and coagulation tests could be performed to exclude any bleeding disorders. In one case, symptoms mimicked an epididymo-orchitis, due to leucocytosis [3]. No ultrasound was performed. The delay in treatment resulted in ischaemia due to obstruction, necrosis of the testis, and eventually orchiectomy.

CUD is the imaging modality of choice in evaluating patients who present with acute scrotal pain [35], especially in cases where testicular blood flow is of the essence. Gray-scale images are nonspecific for detecting testicular torsion. A distinction should be made between intra-testicular and extra-testicular hematomas, since intra-testicular hematomas can result in testicular infection or necrosis in 40% of patients without exploration [35]. A hematoma on CUD is shown as a heterogeneous, hypo-echogenic, non-vascular mass, which can be mistaken with a malignancy. CT-Abdomen or MRI can be used to diagnose arteriovenous malformations.

Imaging of the scrotum was performed in all but four cases [4, 10, 12, 16], where CUD was the mostly used modality. In all four cases without any imaging, exploration was performed, with one resulting in an orchiectomy due to suspected tumour [16].

### Treatment

Surgical exploration is indicated when diagnosis is uncertain, or when a testicular torsion or ischaemia cannot be excluded, and orchiectomy can be performed when malignancy is highly expected, (or partial or a intraoperative biopsy). However, physicians should be cautious for unnecessary orchiectomy. When performing exploration, an inguinal approach is recommended, if malignancy is possible, to avoid scrotal violation. Additional arguments for exploration are possible future complications, i.e. testicular ischaemia due to obstruction of the hematoma on the funiculus [14], possible hematoma infection, or no regression in size of the hematoma. Eventually, microscopic ligation/embolisation of the varicocele can be performed [7, 9].

Overall, exploration was performed in ten patients (56%), indicating the lack of knowledge about idiopathic spontaneous hematomas of the scrotum. Direct exploration at presentation was performed in eight patients (44%). Reasons of direct exploration were uncertain diagnosis (63%) [4, 8, 12, 16, 18], suspected incarcerated inguinal hernia [10], or persisting pain [14, 20]. In the two patients, delayed exploration was performed due to no improvement of symptoms, or ischemia of the left testis [3, 6]. In 25% of patients, direct exploration resulted in orchiectomy [16, 18].

Present varicocele is usually diagnosed with imaging or surgical exploration after the forming of the hematoma. Besides the case report of Gordon et al. 1993 [8], this is the only known case report where the patient had a known untreated varicocele. However, there have been case reports without signs of a varicocele at exploration [4]. In eight case reports, a present varicocele was reported, after either CUD or during exploration. It is not known if special attention was given to possible varicoceles during US examination in all cases.

In general, the scrotal hematoma is considered self-limiting and should be treated conservatively. Symptoms can be treated with NSAID’s, and RICE (rest, ice, compression and elevation of the scrotum).

In our patient, the hematoma increased in size up to 3 months post-event. Only after microscopic inguinal varicocelectomy the size of the hematoma started to decrease. It is unsure whether this is a result of the treatment or time. After 15 months, the hematoma was dissolved completely. It is important to inform the patient of the possible duration of completely reabsorption to avoid anxiety and unnecessary follow-up diagnostics.

Avoiding increased abdominal pressure for patients with known varicocele, to avoid spontaneous ruptures does not seem indicated. The scarcity of case reports, as a complication of a condition present in about 15% of males, shows its rarity. Recurrent hematomas have never been described.

## Conclusion

An acute scrotal hematoma secondary to a spontaneous varicocele rupture is a rare phenomenon. A literature search on case reports shows that clinical presentation is often similar to testicular torsion, appendix testis torsion, or malignancy, making differential diagnosis difficult. Especially when there has been increased abdominal pressure or strenuous activity preceding the symptoms, and the swelling is left sided, it should be included in the differential diagnosis for patient with acute inguinoscrotal swelling. Colour Doppler-Ultrasonography is the golden standard to distinguish between other causes of acute scrotum. Early surgical intervention is justified with signs of ischaemia due to obstruction, infection of the hematoma, or uncertain diagnosis (i.e. malignancy). However, physicians should be cautious whereas direct exploration was performed in almost half the case reports, which led to unnecessary orchiectomy in 25% of patients. Otherwise, the hematoma is self-limiting, and conservative treatment is recommended. The hematoma can increase in size up to 3 months post-event, and it can take up to 15 months for the hematoma to completely resolve.

## Abbreviations

CDU: Colour Doppler-Ultrasonography
